# Obesity in school-aged children and its correlation with Gut *E.coli* and *Bifidobacteria*: a case–control study

**DOI:** 10.1186/s12887-015-0384-x

**Published:** 2015-05-30

**Authors:** Xiaolin Gao, Ruizhen Jia, Liang Xie, Linghan Kuang, Ling Feng, Chaomin Wan

**Affiliations:** Department of Pediatrics, West China University Second Hospital, Sichuan University, 610041 Chengdu, Sichuan China; Open Laboratory, West China Institute for Women’s and Children’s Health, 610041 Chengdu, Sichuan China; Pulmonary Vascular Remodeling Research Unit Laboratory, West China Institute for Women’s and Children’s Health, 610041 Chengdu, Sichuan China; Department of Laboratory Medicine, Sichuan University West China Second Hospital, 610041 Chengdu, Sichuan China

**Keywords:** Obesity, Children, Gut microbes, *E.coli*, and *Bifidobacteria*

## Abstract

**Background:**

To determine the correlation between obesity in school-aged children and imbalance of gut microbes by examining the ratio change of intestinal *Bifidobacteria* and *E.coli* in obese children compared to non-obese controls.

**Methods:**

A hospital-based 1:1 case–control study was performed. Fecal samples of the subjects were collected for DNA extraction and analyzed by quantitative real-time PCR (qPCR) to determine the copy number of *Bifidobacteria* and *E.coli*. The ratio of two microbes (B/E) was then calculated and statistically analyzed.

**Results:**

Subjects of the obesity group and control group showed no significant difference in age, gender or height (*P* > 0.05); whereas they had significant differences in body weight and BMI. Copy numbers of *Bifidobacteria* and *E.coli* per gram of wet fecal samples were first determined using qPCR in both obese and normal groups, which were further used for the calculation of B/E ratio. We found that B/E ration in the two groups showed significant difference (*P* < 0.05). Corrected χ^2^ test was performed for the two groups against B/E < 1, and it was found that there was a positive correlation (*OR* = 719.2, *OR 95% C.I.* = 81.57-6341.18) between B/E ratio decrease with childhood obesity.

**Conclusions:**

The obese children have a lower amount of *Bifidobacteria* and higher amount of *E.coli* (smaller B/E ratio) compared to normal non-obese children. It was suggested that obesity in children may be associated with the imbalance of gut microbes.

## Background

Obesity is the excessive accumulation of fat all over the body due to a variety of factors. The impact of obesity on metabolism, endocrine, function of the cardiovascular, respiratory and digestive systems as well as on the growth and development of the body has been well studied [1]. Childhood obesity is closely correlated with adult obesity, diabetes, hyperlipidemia, hypertension and coronary heart disease. In recent years, with the improvement in people’s living condition and the changes in diet and other factors, childhood obesity is increasingly prevalent and occurs in younger children [2,3]. Among the non-genetic risk factors of obesity, the role of gut microbes has been recognized. The correlation between obesity and changes in gut microbes has been demonstrated in multiple studies using animal models of obesity, diabetes and other diseases [4-6]. In addition, it has been shown that E. coli numbers were higher in women with excessive weight gain than in women with normal weight gain during pregnancy. In contrast, Bifidobacterium and Akkermansia muciniphila showed an opposite trend [7]. Thus far, the correlation between obesity in children and gut microbes has been scarcely well studied, and the significance of the ratio of intestinal *Bifidobacteria* and *E.coli* (B/E ratio) remains to be defined. The current hospital-based 1:1 case–control study examined the ratio changes of intestinal *Bifidobacteria* and *E.coli* in obese and non-obese school-aged children using quantitative real-time PCR. Our data indicate that there was a positive correlation between obesity and imbalance of gut microbes.

## Methods

### Subjects and study groups

This study has been conducted with written approval from the ethics committee of West China University Second Hospital and written consent from both subjects and their parents/guardians.

This study was a hospital-based 1:1 case–control with random sampling. Inclusion criteria included: obese group subjects were recruited from children visited Child Health Clinic of West China University Second Hospital from February 2012 to June 2012 and diagnosed with obesity according to the WHO/NCHS standard [8,9]; and non-obese control subjects were recruited from these with a record of the annual health examination during the same period and in the same hospital without a diagnosis of obesity. The obese and control children were paired according to age and gender. Exclusion criteria included antibiotics or probiotics receiving, or a history of diarrhea or other gastrointestinal diseases in the past 4 weeks; heart, lung, liver, kidney, endocrine, genetic and metabolic diseases; or unwillingness to participate in this study.

Age, gender and other general information of the subjects were recorded; height (m) and weight (kg) of subjects of the two groups were measured; fecal samples of all subjects were collected with sterile and sealed box within two hours after defecation, and immediately stored at −70°C for further preparation.

### Experimental methods

The experimental procedure included: preparation of standard stains (revival, culture, passage, storage and DNA extraction), extraction of DNA from fecal samples, determination of DNA concentration and purity, primer design for detection of specific microbe strains, establishment of standard curve with standard strain DNA, and Quantitative real-time PCR (qPCR) analysis of the samples, which is was carried out following manufacturer’s instructions.

The primers were synthesized by TaKaRa. Primer sequences of forward (F) and reverse (R) used in this study are as follows. F: 5′-TTGGGCGTAAAGGGCTCGTA-3′, and R: 5′-TTCGCCATCGGTGTTCTTCC-3′ for *Bifidobacteria*, which produce a PCR product with the length of 166 bp. F: 5′-GTTAATACCTTTGCTCATTGA-3′ and R: 5′-ACCAGGGTATCTTAATCCTGTT-3′for *E.coli* with a 144 bp PCR product.

Quantitative fluorescence PCR analysis of the samples: sample DNA was extracted, and amount of *Bifidobacteria* and *E.coli* 16 s rRNA was determined by quantitative fluorescence PCR analysis using the same reaction system and conditions as establishment of the standard curve. Ten-fold serial dilutions of the standard and fecal bacteria DNA samples were co-currently tested. Standard and blank controls were included in each experiment, and each sample was tested in triplicate. Specificity of the PCR reaction was confirmed by the melting curve, and copy number of the bacterial gene was calculated using Ct value and the standard curve. Data were presented as the logarithm of copy number ($\overline{x}$*lgx ± slgx*), and ratio of *Bifidobacteria* to *E.coli* (B/E) was calculated.

### Statistical analysis

Data were analyzed using the SPSS software package. Quantitative data were presented as mean ± standard deviation ($\overline{x}$*± sd*). Comparison was performed using two-tailed *t*-test. Categorical data were compared using 2 × 2-table χ^2^ test (Yates’ χ^2^ test or Fisher exact test was used when the criteria were not met). Odd Ration (*OR)* value and *95 % C.I.* were calculated. *P* < 0.05 was considered statistically significant.

## Results

General information: both obesity group and control group included 63 subjects (Table 1). Two groups showed no significant difference in age, gender and height (*P* > 0.05), whereas they had significant difference in body weight and BMI (*P* < 0.05).

**Table 1:** The general information of subjects ($\overline{x}$ *±s, n*).

	Obesity (*n* = 63)	Control (*n* = 63)
Male	32	31
Female	32	31
Age	6.8 ± 2.1	6.8 ± 2.4
Height (m)	1.25 ± 0.11	1.20 ± 0.15
Weight (kg)	35.4 ± 5.8	24.0 ± 2.3
BMI (kg/m^2^)*	23.2 ± 5.2	15.2 ± 1.6

*BMI (BodyMass Index) = weight (kg)/height^2^ (m^2^).

Determination of DNA concentration and purity: A260/A280 value of standard DNA was 1.83 ± 0.17 for *Bifidobacteria* and 1.86 ± 0.13 for *E.coli*; A260/A280 value of the extracted DNA was 1.92 ± 0.28 for the obesity group and 1.89 ± 0.26 for the control group. No significant difference of DNA concentration and purity was detected between the two groups (*P* > 0.05).

**Fig. 1:**
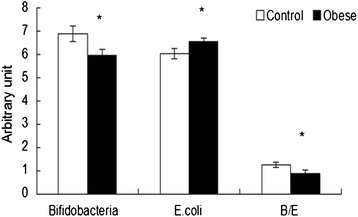
Quantitative real-time fluorescent PCR results for fecal specimen DNA content of *Bifidobacteria* and *E.coli* ($\overline{x}$ *lgx ± slgx,*copy number/g) and their ratio in obese and normal subjects. (*, *P* < 0.05)

Quantitative real-time PCR results of the fecal samples (Fig. 1, data were presented as the logarithm of copy number ($\overline{x}$*lgx ± slgx*).

Based on whether the B/E ratio value is lower than 1, distribution of subjects of the two groups were filled into a 2 × 2 table (Table 2). Because *n* > 40 and 1 ≤ *T* < 5,Yates’ χ*2* test was used. The results showed χ^2^ = 99.96, γ = 1, *P* < 0.0001, indicating a significant difference between obesity and control groups; Our further calculation indicated *OR* = 719.2 with a *95%C.I*. of 81.57 ~ 6341.18. It was suggested that there is a positive correlation between obesity and B/E < 1.

**Table 2:** The B/E ratio distribution in obese and normal groups *n (%)*

	B/E ratio < 1	B/E ratio ≥ 1	Total
Obesity	58 (92.06)	5 (7.94)	63
Control	1 (1.59)	62 ((98.41)	63
Total	59	67	126

## Discussion

As reported by WHO in recent past years, childhood obesity is rapidly increasing globally and the incidence of obesity has doubled since 1980, and over 40 million children are currently living with obesity [9]. While the pathogenesis of obesity is not clearly defined, the roles of gut microbes has been proposed [1,10]. In a rat model, it has been demonstrated that obesity can be transferred by gut microbes: rats received gut microbes from obese donor showed obesity but not slim donor [11]. Accumulating data in the literature support the idea that gut microbes, the largest and the most important micro-ecological system in human body, plays a significant role in health. Under normal circumstances, bacteria in the intestine maintain a certain proportion of species and amount to sustain the stability of intestinal micro-ecology. However, such stability could be disturbed by a variety of factors, including metabolism, immune system as well as the inflammatory reactions of the host, and subsequently results in obesity and other diseases [12-14]. In the mean time, gut microbiota is under influence of physiological conditions, diet and medication. For example, antibiotics [15], pregnancy [16] or by probiotics [17,18] causes weight gain through modulating gut flora. However, very limited data are available in the literature regarding gut microbes and childhood obesity. It becomes particularly important to study relationship between gut microbes and obesity because of the rapid increase of childhood obesity. The traditional method for identification and quantification of fecal bacteria is culturing approaches, which is time- and labor-consuming, and the results may vary due to different operations. With the advance of molecular biological techniques, culture-free methods have emerged. Currently, the 16 s rRNA-based qPCR is widely used in micro-flora studies because of its high accuracy and sensitivity as well as low chance of contamination [19,20].

In the current hospital-based 1:1 case–control study, fecal DNA were prepared from obese and normal control subjects and examined along with the DNA of standard stains. A260/A280 of these DNA was determined to be 1.6-1.8, indicating good quality of the extracted DNA. Standard DNA was prepared using standard strains and tested by conventional PCR and quantitative fluorescent real-time PCR to establish the standard curve. Primers had good specificity for examined stains, as indicated by results of conventional PCR and melting curve. Quantitative fluorescent real-time PCR was further used to quantify intestinal *Bifidobacteria* and *E.coli* content, and the results showed that the obese children had significantly lower content of *Bifidobacteria* and higher content of *E.coli*, compared to normal control. It was suggested that imbalance of gut microbes was present in these obese children. Ratio of intestinal *Bifidobacteria* and *E.coli* (B/E) was first introduced by Dutch scholar Van Der Waaij et al. [21] to represent colonization resistance of gut microbes. *Bifidobacteria* is a typical beneficial bacterium of the intestine, while *E.coli*, a representative pathogenic bacterium. Decrease in B/E is considered as an important indicator for the shifting from normal gut microbes to an adverse condition to health. Because these two types of bacteria are also normal part of gut microbe flora, the B/E ratio represents the overall condition of intestinal micro-flora [22]. In this study, B/E ratio was significantly lower in obese children than in normal controls, and there was a strong correlation between obesity and B/E < 1, which are consistent with the studies conducted by Million [18] and Santacruz [7] in animal model and other populations. Taken together, lower *Bifidobacteria* colonization and imbalance of gut microbes may play an important role in the development of obesity.

## Conclusions

In summary, the correlation between obesity and gut microbes has been one of the hottest research topics. Studies on gut microbes have achieved substantial advances using animal models. More investigations are necessary to clearly defined its roles in human obesity. The B/E ratio is a widely used parameter for estimating the overall condition of gut flora. However, gut microbes consist of large and complex system. Further study for the whole spectrum of their functions and mechanisms in healthy and pathological conditions may provide therapeutic targets of the treatment of obesity.

## Abbreviations

BMI: Body Mass Index
DNA: Deoxyribonucleic acid
PCR: Polymerase chain reaction
B/E: Ratio of *Bifidobacteria* and *E.coli*
